# Common Data Elements Regarding Social Determinants of Health in Pediatric Epilepsy Research: A Concept Mapping Study

**DOI:** 10.1016/j.pediatrneurol.2025.01.025

**Published:** 2025-02-06

**Authors:** Laura Kirkpatrick, Chethan K. Rao, Christopher W. Beatty, Sonal Bhatia, Charuta Joshi, Kristina Julich, Shital H. Patel, Rachit Patil, Janelle L. Wagner, Christina Briscoe Abath, Alexandria Melendez-Zaidi, Sara E. Baumann, Jessica G. Burke, Qian-Zhou JoJo Yang

**Affiliations:** a Department of Pediatrics, University of Pittsburgh School of Medicine, Pittsburgh, Pennsylvania; b Department of Pediatrics, University of Maryland School of Medicine, Baltimore, Maryland; c Department of Pediatrics, The Ohio State University and Nationwide Children’s Hospital, Columbus, Ohio; d Department of Pediatrics, Medical University of South Carolina, Charleston, South Carolina; e Department of Pediatrics, Children’s Medical Center/UT Southwestern Medical Center, Dallas, Texas; f Department of Neurology, The University of Texas at Austin Dell Medical School, Austin, Texas; g Department of Pediatrics, Duke University, Durham, North Carolina; h Department of Pediatrics, Alpert Medical School of Brown University/Hasbro Children’s Hospital, Providence, Rhode Island; i College of Nursing & Comprehensive Epilepsy Center, Medical University of South Carolina, Charleston, South Carolina; j Department of Neurology, Boston Children’s Hospital, Boston, Massachusetts; k Department of Neurology, Texas Tech University Health Sciences Center, El Paso, Texas; l University of Pittsburgh School of Public Health, Pennsylvania; m Department of Neurology, University of North Carolina at Chapel Hill, Chapel Hill, North Carolina

**Keywords:** Epilepsy, Common Data Elements, Health equity, Health disparities, Pediatric epilepsy, Social determinants of health

## Abstract

**Background:**

The Pediatric Epilepsy Research Consortium (PERC) Health Equity Special Interest Group (SIG) used Concept Mapping to begin developing Common Data Elements (CDE) about social determinants of health to standardize data collection and facilitate robust evaluation of health disparities in pediatric epilepsy research.

**Methods:**

Concept Mapping is a structured participatory mixed method suited for developing group consensus. PERC members (1) identified social factors that are important to measure in pediatric epilepsy research, (2) sorted factors into meaningful categories, and (3) rated the factors on importance and ease of measurement. The authors applied multidimensional scaling to the sorting data, created spatial point maps, and used hierarchical cluster analysis to define concepts. A bivariate scatterplot was used to explore importance versus ease of measurement to prioritize factors for inclusion. The PERC Health Equity SIG met on three occasions to interpret research findings and finalize a CDE set.

**Results:**

Eighty-one PERC members generated 110 candidate factors. Thirty PERC members completed sorting, and 48 completed rating. The factors grouped into a five-cluster solution: “Household and Neighborhood Resources,” “Family Context,” “Individual Demographics,” “Healthcare Experiences,” and “School.” Sixty-two items were rated with high importance, of which 34 were rated with high ease. The PERC Health Equity SIG decided by consensus to include most items with high importance and high ease ratings, plus selected additional items. The final CDE set consists of 42 items.

**Conclusions:**

Inclusion of CDE in future pediatric epilepsy research will enable researchers to undertake systematic analyses of health disparities.

## Introduction

Available limited data suggest that health disparities may be common in pediatric epilepsy in the United States.^1^ Despite limitations of the data, disparities have been identified in key outcomes such as mortality and quality of life with differences by demographic variables such as sex, race and ethnicity, and socioeconomic status.^1^ However, existing research is limited by poor collection and reporting of sociodemographic variables, which may undermine current understanding of health disparities in pediatric epilepsy.^1^

The Health Equity Special Interest Group (SIG) of the Pediatric Epilepsy Research Consortium (PERC) recently conducted a scoping review of the literature to enhance understanding of health disparities in pediatric epilepsy in the United States.^1^ The scoping review identified that factors such as education, geography, language, and immigration status were notably under-represented in the published literature.^1^ These findings underscore the need for broader inclusion and representation of diverse sociodemographic factors in future research efforts to comprehensively understand health disparities in pediatric epilepsy.^1^

In response to these findings, the PERC Health Equity SIG aimed to develop a set of Common Data Elements (CDE) for social determinants of health for inclusion in pediatric epilepsy research. The objective for developing these CDE is to promote standardized data collection to better facilitate identification and analyses of health disparities in pediatric epilepsy. Novel CDE focusing on social determinants of health are needed as existing CDE sets for epilepsy research have not prioritized health equity variables as a core content area of analysis or are not specifically tailored to pediatric epilepsy in the US context.^2–6^

In this study, we used Concept Mapping methodology to develop a CDE set in collaboration with PERC members.^7–10^ Concept Mapping is a structured participatory methodology, with both qualitative and quantitative components, which aims to generate consensus among collaborators around a topic. Drawing upon the collective expertise of PERC in developing the CDE increases the likelihood that the included variables are both highly relevant to pediatric epilepsy research and practical for implementation in research studies by investigative teams. Future steps will include refining the CDE with input from youth with epilepsy and caregivers, as well as PERC membership.

The development of these CDE will allow for comprehensive analysis of health equity outcomes, even in studies that are not primarily focused on disparities. Systematic examination of the diverse dimensions of health equity and their impact on pediatric epilepsy outcomes within studies can inform how to address gaps in clinical practice, policy changes, and priorities for future health disparities research and interventions.

## Materials and Methods

### Concept Mapping

For this project Concept Mapping was implemented in three phases: (1) brainstorming, (2) sorting and rating, and (3) interpretation sessions for contextualizing and finalizing the study findings.^7^ In the brainstorming phase, participants generate a list of ideas related to a prompt/question. In the sorting and rating phase, participants receive the list of ideas generated during brainstorming, which they sort into conceptual categories based on their own understanding, experience, and interpretation of the topic. Next, participants rate each item on the list according to predetermined scales. In the third phase, study team members lead a facilitated discussion among participants about the study findings to interpret and finalize the results. In this study, members of the PERC Health Equity SIG used groupwisdom software (Ithaca, NY, USA) to collect the data online, manage the data, and perform the data analysis.

### Sample size in Concept Mapping

Owing to its incorporation of qualitative methodology, sample size in Concept Mapping is based on achieving data saturation rather than quantitative power.^7–10^ Data saturation occurs when no new information is generated through further sampling.^11^ We set a minimum goal of 15 participants per activity based on prior literature on sufficient sample size for Concept Mapping and with input from coinvestigators (J.G.B. and S.B.) who are leading experts in Concept Mapping.8 Participants were required to be members of PERC.

### Question and prompt development

Adopting a collaborative approach, members of the PERC Health Equity SIG developed all questions and prompts for data collection. Question format and language was discussed and refined in teleconference meetings, with further opportunity for asynchronous revision over e-mail.

### Phase 1: Demographics and brainstorming

We opened a demographics survey and the brainstorming activity from March 2024 through April 2024. Participants were able to complete these activities asynchronously and anonymously. The PERC Executive Director invited members to complete these activities through e-mails to the entire PERC membership (n = 242), e-mails to PERC members registered for an in-person annual meeting in May 2024, and announcements in the PERC monthly newsletters. Members of the PERC Health Equity SIG also promoted the study to each of PERC’s other 12 SIGs either by attending virtual meetings or by sending an e-mail to the group. The other 12 epilepsy SIGs concern Behavioral Health, Myoclonic Atonic Seizures, Infantile Spasms, Surgery, Neuromodulation, Telehealth, Early Life Epilepsy, Lennox-Gastaut Syndrome, Psychogenic Non-epileptic Seizures, Genetics, and Developmental and Epileptic Encephalopathy with Spike-Wave Activation in Sleep.

Participants were asked to view a screen of information about the study and click a link to provide informed consent. For demographics, we asked participants to answer five multiple choice questions pertaining to their profession, years in practice post-training, gender identity, race, and ethnicity.

For brainstorming, we asked the prompt: “In your opinion, what are the important social factors to measure and report when doing pediatric epilepsy research? Please list all.” Participants answered this question with free text entry. Participants were able to view all previous responses from other participants as well.

### Phase 2: Sorting and rating

Before opening the sorting and rating activities, the principal investigator (L.K.) reviewed all responses to the brainstorming prompt and collated a list to disaggregate multiple factors included in individual responses and remove duplicate items. Members of the PERC Health Equity SIG reviewed the original list of responses and proposed any changes as needed to the collated list to ensure fidelity to the original responses.

The sorting and rating activities were conducted in person at the annual PERC meeting in May 2024. We invited meeting attendees to complete the activities online using a personal electronic device such as a laptop, tablet, or phone individually and anonymously during a one-hour session. Although encouraged, participation was voluntary. The online activities remained open for one week following the meeting if more time was needed to complete them. PERC members who were not able to attend the meeting were also e-mailed an invitation to complete the activities online during that one-week period. Members were encouraged, if they had limited time, to prioritize completing the rating activity over the sorting activity due to the lower time burden of rating.

Before sorting and rating, participants were again asked to view a screen of information about the study and click a link to provide informed consent. Because participation in brainstorming (the first phase) was not required to participate in the sorting and rating phase, the demographics survey was also repeated.

For the sorting activity, participants were instructed to “Group the statements on how similar in meaning they are to one another.” Participants were able to drag and drop the individual statements into piles in the online software and were prompted to provide a name for each pile reflecting the theme of each pile.

For the rating activity, participants were asked to rate each individual item on two five-point Likert scales for importance and ease of measurement. First, they were asked: “In pediatric epilepsy research, how important is it to measure this factor to assess health disparities? Please rate your answer on a scale from 1-not at all important to 5-extremely important. Please try to use the full rating scale.” Second, they were asked: “In pediatric epilepsy research, how easy would it be for investigators to measure this factor in their studies? Please rate your answer on a scale from 1-extremely difficult to 5-extremely easy. Please try to use the full rating scale.”

### Data analysis

We applied multidimensional scaling to the sorting data and created spatial point maps, which is a standard analysis technique for Concept Mapping data. In the spatial point map, each item is represented on the map by a point and the relative distance between two points reflects how frequently the items were sorted together, suggesting degree of conceptual similarity or difference. We used hierarchical cluster analysis to define concepts (thematically related groups of items). The analysis generates multiple potential cluster solutions. In this study, the research team selected the cluster solution that appeared to best fit the data. We used the rating data to create a “Go-Zone plot”—a bivariate scatterplot of importance versus ease of measurement to prioritize factors for inclusion in the CDE set. The “Go-Zone plot” is divided into quadrants of “high importance and high ease”; “high importance, low ease”; “low importance, high ease”; and “low importance, low ease.” It is important to note that the terms “low importance” and “high importance” are relative to each other within this analysis and do not reflect the absolute importance of these concepts to health disparities. We also calculated the Pearson correlation co-efficient between importance and ease ratings.

### Phase 3: Interpretation and finalization meetings

The PERC Health Equity SIG met on three occasions by teleconference in May and June 2024 to interpret research findings and finalize a CDE set. Attendees were not required to have previously participated in either brainstorming or sorting and rating activities. Through discussion facilitated by the principal investigator (L.K.) and senior investigator (J.Y.), attendees reviewed the spatial point maps and finalized conceptual categories including agreeing upon names/labels for each cluster. We used the “rating” data to prioritize items for inclusion. We used the “sorting” data to ensure representation of items from each thematic cluster. We used the quadrants within the “Go-Zone plot” to guide selection of CDE. The PERC members decided by consensus, based on the findings, which items to include in the CDE set based on the data. The PERC members also decided whether items should be included in both retrospective and prospective studies versus prospective studies only (as some items might not be feasibly available for retrospective data collection from typical data sources like the electronic medical record or administrative claims). We achieved consensus through facilitated discussion.

### Ethical approval

The University of Pittsburgh Institutional Review Board deemed this study to be exempt from full review.

### Data availability

Data from this study are available upon request to any interested investigator.

## Results

### Participants

Eighty-one PERC members completed brainstorming, 30 PERC members completed sorting, and 48 PERC members completed rating activities. Therefore, we reached the target of at least 15 participants per activity. These numbers of participants represent robust samples for Concept Mapping.^5,7^ From the 242 individuals on the PERC e-mail listserv, 33% participated in brainstorming, 12% in sorting, and 20% in rating. These response rates are in line with those typical in the literature for health care professional surveys.^12^

Detailed participant demographics for each Concept Mapping activity are available in Table 1. Male participants across activities ranged from 30% to 37%; 56% to 63% identified as white and 90% to 97% non-Hispanic, with 65% to 70% of participants reporting their profession as being a physician in pediatric neurology or epileptology.

### Brainstorming

Participants generated 181 items in the brainstorming activity. After removal of duplicates and collation of the list, there were 110 distinct items included for sorting and rating activities. Some examples of items included “neighborhood-level socioeconomic indices,” “whether patient is technology-dependent (i.e., has a tracheostomy or G-tube),” and “access to transportation.”

### Sorting

Based on sorting data, we generated a spatial point map. The stress value for the spatial point map was 0.289; this reflects the goodness of fit of the spatial point map to the sorting data, with lower values indicating better fit and values up to 0.289 indicating that the visual map accurately depicts the quantitative data and is a good fit.^13^ Most Concept Mapping projects have stress values between 0.205 and 0.365 (mean of 0.285).^7^

**FIGURE 1.**
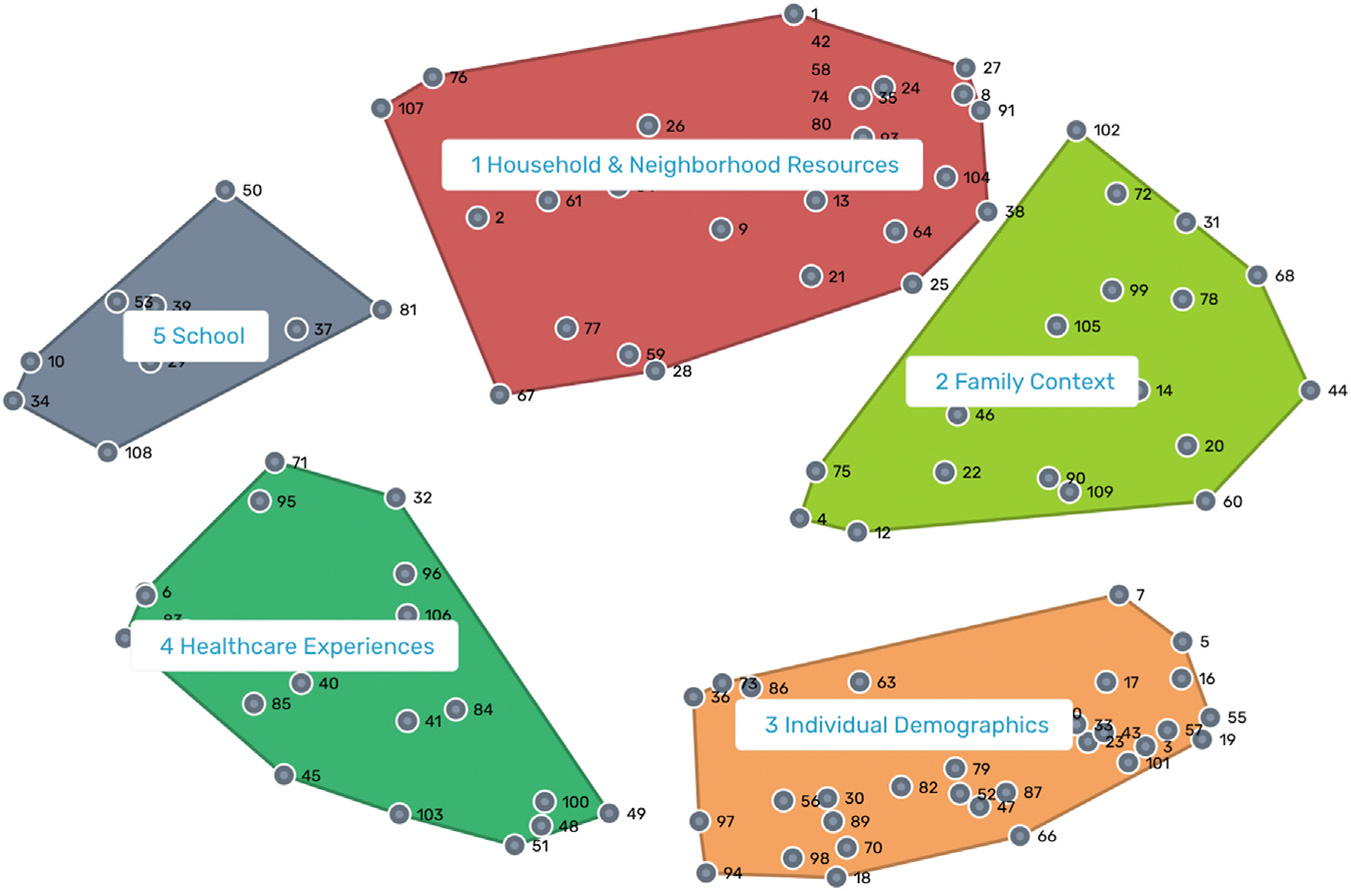
Cluster map. Each point on the map reflects a statement from the brainstorming data. The position of each point, including its distance from other points, reflects its relative similarity or difference from each other point based on the sorting data. The points were grouped into clusters based on the sorting data. The clusters were labeled in the interpretation session. The color version of this figure is available in the online edition.

After applying hierarchical cluster analysis to the spatial point map, the research team selected a five-cluster solution. The PERC Health Equity SIG named each cluster based on its thematic contents, reviewing the items within each cluster and the list of candidate labels provided by the participants in sorting and rating. The final names were “Household and Neighborhood Resources,” “Family Context,” “Individual Demographics,” “Healthcare Experiences,” and “School.” Example items in “Household and Neighborhood Resources” include “Household food security” and “Neighborhood safety.” Example items in “Family Context” include “Whether patient is in foster care” and “Household income.” Example items in “Individual Demographics” include “Ethnicity of patient” and “Preferred language of parent(s)/caregiver(s).” Example items in “Healthcare Experiences” include “Whether and how much parent(s)/caregiver(s) trust healthcare professionals” and “Patient experiencing racism or other ostracizing situations in healthcare or from providers.” Example items in “School” include “Whether patient is in school” and “Whether patient has an Individualized Education Plan.” The cluster map, superimposed on the spatial point map, is displayed in Fig 1.

### Rating

**FIGURE 2.**
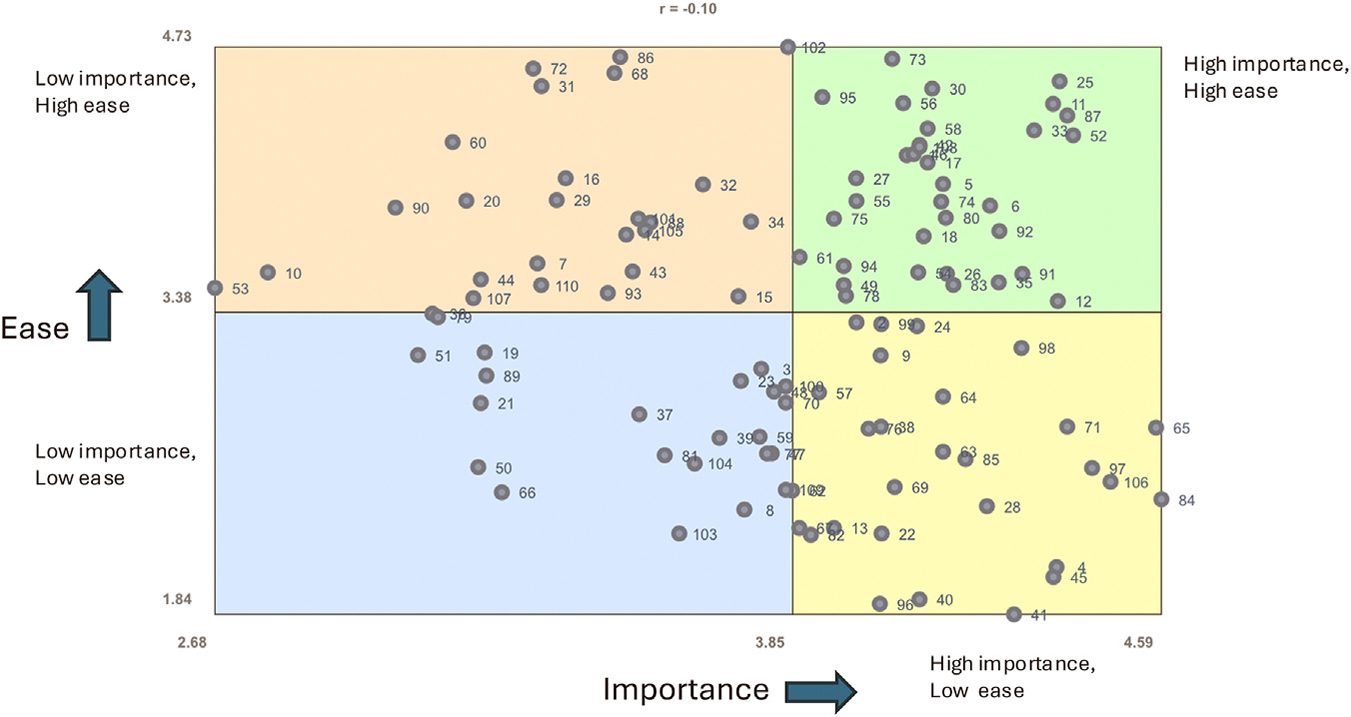
Go-Zone plot. Each point on the plot reflects a statement from the brainstorming data. The x axis reflects importance ratings from 0 to 5. The y axis reflects ease of measurement ratings from 0 to 5. The plot is divided into quadrants with the upper right-hand quadrant reflecting high importance and high ease. The color version of this figure is available in the online edition.

**FIGURE 3.**
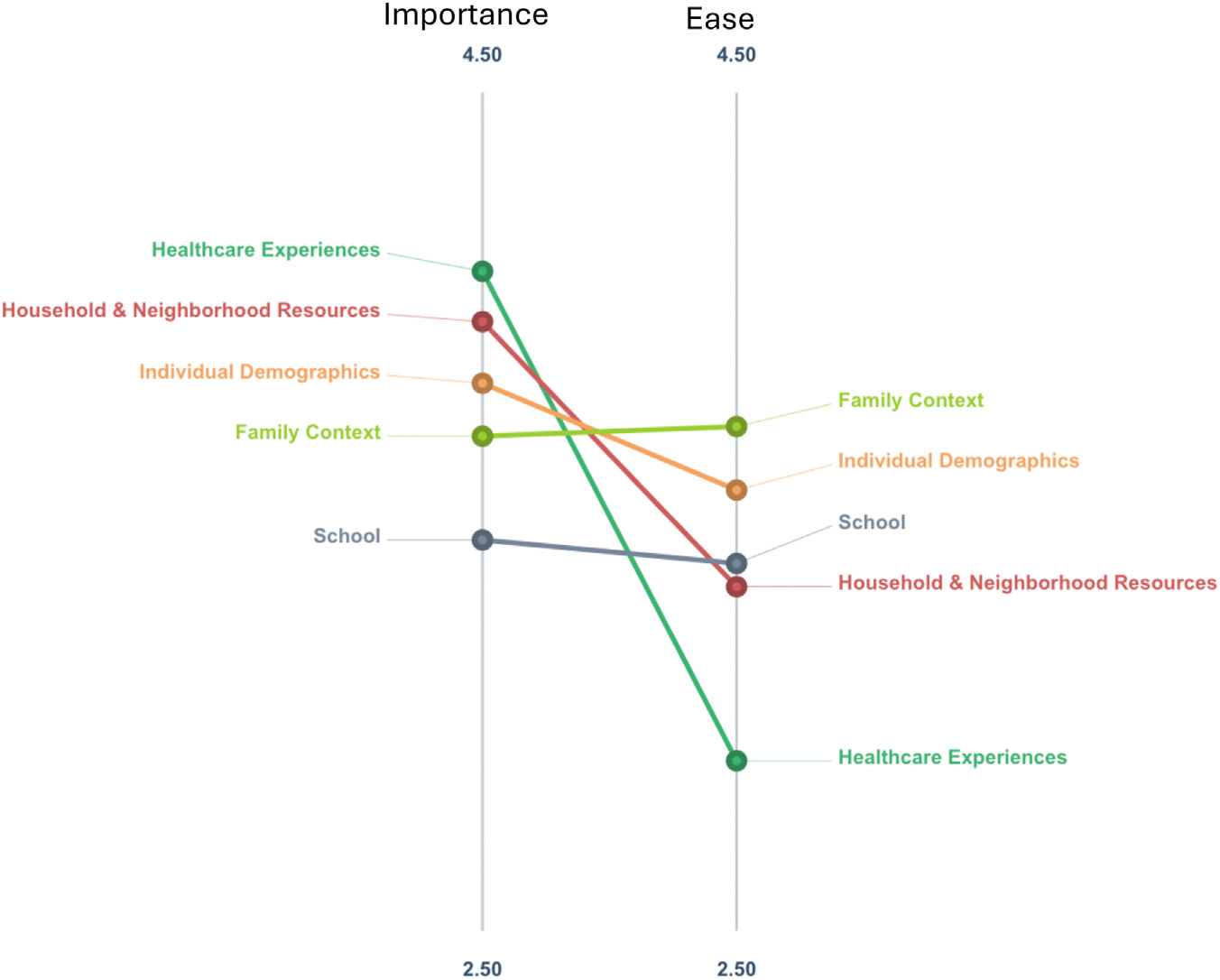
Pattern matching display of relative importance and ease of measurement ratings for clusters. Each cluster is represented by two points connected by a line. The point on the left reflects its cluster-level rating of importance. The point on the right reflects its cluster-level rating on ease of measurement. The color version of this figure is available in the online edition.

For individual items, ratings for importance ranged from 2.68 of 5 (lowest rated item: “Patient participation in after-school sports”) to 4.59 of 5 (highest rated item: “Parent(s)/caregiver(s) understanding of the diagnosis/prognosis/treatment of epilepsy”). Ratings for ease of measurement ranged from 1.84 (lowest rated item: “Patient experiencing racism or other ostracizing situations in healthcare or from providers”) to 4.73 (highest rated item: “Zip code”). There was a weak negative correlation between importance and ease of measurement (r = −0.10, Pearson correlation coefficient). The Go-Zone plot for ratings of individual items is displayed in Fig 2. The upper right-hand quadrant of the Go-Zone plot includes items with the highest ratings for importance and ease of measurement. On a cluster level, mean importance and ease of measurement ratings for items in each cluster are displayed in Fig 3.

### Interpretation and finalization

Three interpretation meetings were each attended by 16 individuals from the PERC Health Equity SIG. We decided as a group to include most items in the Go-Zone plot quadrant of “high importance, high ease of measurement” due to the high ratings received by these items. However, we discussed exclusion of select items to avoid redundancy and ensure the group agreed that all items were in fact important and easy to measure based on face validity. For example, from this quadrant, there were seven items pertaining to distance from medical resources. We excluded five of these to avoid redundancy. The group also excluded an item on “spoken English proficiency of patient” due to concerns that defining and measuring what is meant by “proficiency” might not be feasible to measure despite the high ease of measurement rating for this item.

Next, we reviewed the other quadrants of the Go-Zone plot to explore items for potential inclusion despite ratings of lower importance and/or ease of measurement. We did so to ensure that no items deemed to be critical based on the SIG’s expertise were inadvertently omitted.

From the “low importance, high ease of measurement” quadrant, we included six items in the final CDE. The group believed that two of these items were essential for understanding demographics in any study: “sex” and “gender identity.” “Zip code” was included due to the group’s belief that this information would be needed to feasibly determine certain “high importance, high ease” items pertaining to geography. Finally, we included three items from this category to ensure that at least some of the more important items were represented from the “School” cluster: “if the patient has a 504 plan,” “if the patient has an Individualized Education Plan,” and “if the patient is involved with Early Intervention services.” Notably no items from the “School” cluster were located in the “high importance, high ease” quadrant.

From the “low importance, low ease of measurement” quadrant, we included two items in the final CDE. These included “patient legal status in the USA” and “sexual orientation/sexual identity of patient.” The group believed that these items were important due to the known existence of health disparities related to immigration status and sexual minority status.^14–17^

From the “high importance, low ease of measurement” quadrant, we included five items in the final CDE. These included “access to transportation, “family quality of life,” “household food security,” “parent/caregiver legal status in the USA,” and “patient/family housing status and security.” The group considered these items sufficiently important to attempt evaluation of these items to understand health equity despite measurement challenges. Notably, due to the perceived high difficulty of measuring these items particularly in retrospective studies, all items included from this quadrant were designated as recommended for prospective studies only.

**FIGURE 4.**
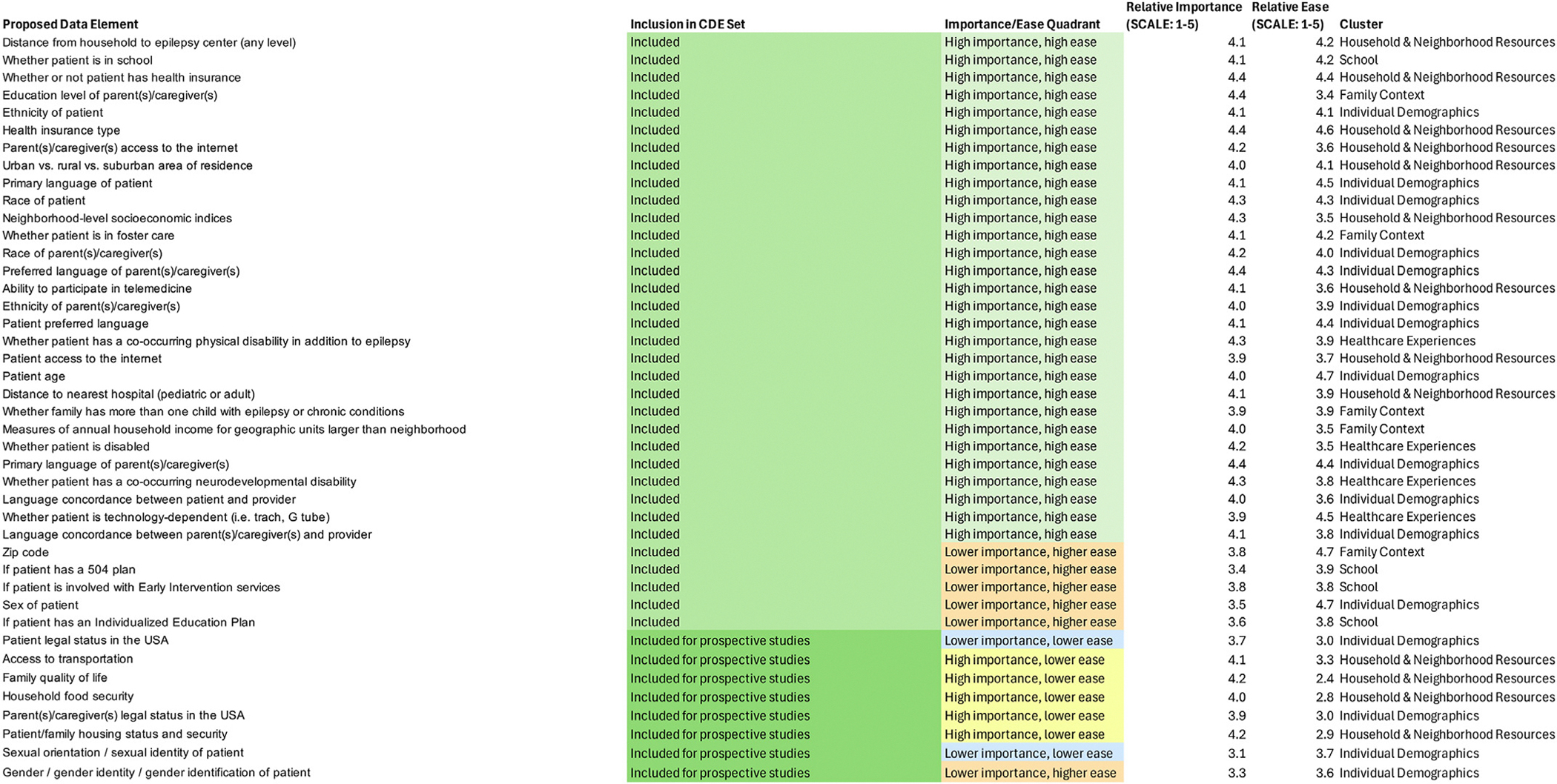
Candidate social factors included in Common Data Elements. This figure displays the proposed data element, its importance/ease quadrant on the Go-Zone plot, its importance rating, its ease of measurement rating, and its cluster. The color version of this figure is available in the online edition.

**FIGURE 5.**
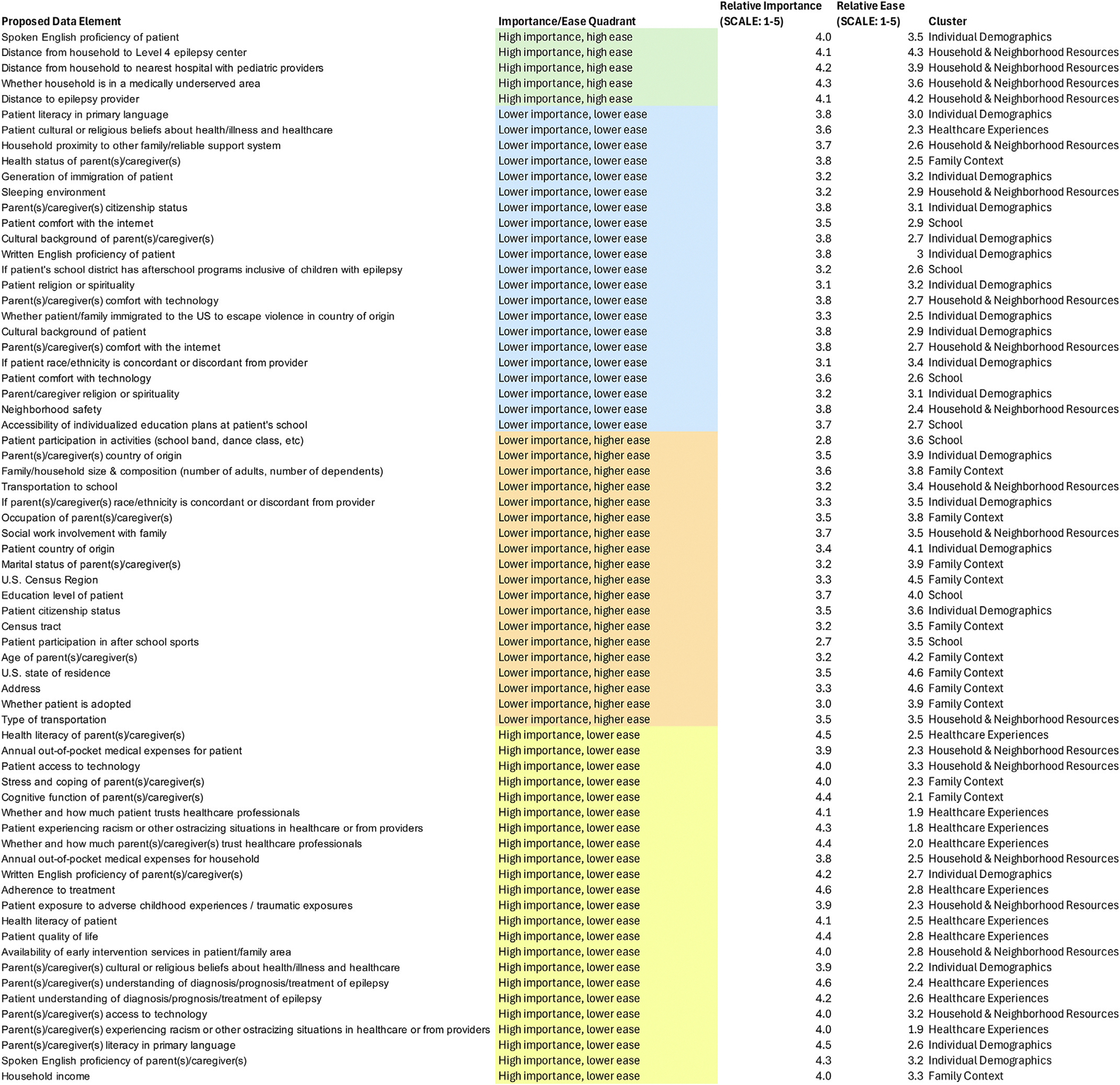
Candidate social factors omitted from the Common Data Elements. This figure displays proposed data elements that were ultimately not included in the Common Data Element set, each item’s importance/ease quadrant on the Go-Zone plot, its importance rating, its ease of measurement rating, and its cluster. The color version of this figure is available in the online edition.

The list of items included in the proposed CDE set, with associated importance and ease of measurement ratings, cluster, Go-Zone quadrant, and inclusion/exclusion from the CDE, is detailed in Fig 4. A list of the omitted items, with similar features detailed, is displayed in Fig 5. The proposed CDE set, comprising 42 items, is displayed in Table 2.

## Discussion

In this study, we used Concept Mapping to draw upon the collective expertise of PERC members with the goal of developing a proposed set of CDE to facilitate consistent identification and evaluation of health disparities in pediatric epilepsy research studies. We did so because a recent scoping review of the pediatric epilepsy literature identified that health disparities in pediatric epilepsy are common, yet important sociodemographic variables are often under-reported in the literature, and this under-reporting may hinder identification and monitoring of health disparities and may lead to erroneous interpretations of existing data.^1^ In our study, the Concept Mapping methodology identified 110 possible items for consideration, which were then sorted thematically and rated on importance and ease of measurement. After considering thematic representation and importance/ease scores, we developed the proposed set of CDE. Next steps will include paring down, prioritizing, and refining the CDE with input from youth with epilepsy, caregivers, and PERC members to develop a refined CDE set that we will recommend for inclusion in pediatric epilepsy research.

These CDE address gaps in existing CDE sets pertaining to pediatric epilepsy by providing a comprehensive set of sociodemographic variables for consistent and high priority identification of health disparities. For example, the Pediatric Epilepsy Learning Health System developed a set of clinical CDE incorporated in the electronic medical record of participating centers.^2^ This set of clinical CDE includes nine domains (diagnosis of epilepsy, seizure frequency, quality of life, epilepsy history, epilepsy etiology, comorbidities, treatment, process measures, and optional longitudinal history notes), none of which pertains to patient/family sociodemographic variables or health equity.^2^ By contrast, our CDE address “Household and Neighborhood Resources,” “Family Context,” “Individual Demographics,” “Healthcare Experiences,” and “School.”

In addition, the National Institutes of Neurological Diseases and Stroke also developed a core CDE set for epilepsy clinical research that includes some sociodemographic variables such as race and ethnicity.^3^ However, most sociodemographic variables in this set are considered supplemental, such as primary language and insurance type.^3^ These variables are also not specifically tailored to the pediatric context. For example, this data element set does not consider that the caregiver’s primary language may not be the same as the patient’s primary language, which may have implications for quality of care.^3^ By contrast, the National Institutes of Health PhenX Social Determinants of Health Assessments Collection includes comprehensive social determinants of health variables for inclusion across studies, although it does not include variables specific to epilepsy, let alone pediatric epilepsy.^5^

The International Consortium for Health Outcomes Measurement Consensus Recommendations recently developed a standard set of outcomes and measurement methods for children with epilepsy, which includes 10 sociodemographic variables.^4^ However, this set is intended for use across all nations and is therefore not specifically tailored to a US context.^4^ Since social determinants of health and health inequities are rooted in local/national contextual factors, a more detailed set of data elements is needed to understand health disparities in the specific US context.^4^ The Epilepsy Learning Health System, composed of both pediatric and adult epilepsy centers, has also recently endeavored to standardize data collection among participating institutions.^6^ However, initial published standardized data metrics from 2021 focused on collection of quality improvement outcomes rather than demographics and social determinants of health.^6^

Next steps for development and implementation of the current CDE set include focus group discussions with PERC leadership about the feasibility of, and barriers and facilitators to, implementation of the CDE across future PERC studies. We will also conduct qualitative interviews with youth with epilepsy and caregivers about the comprehensiveness of the data set to ensure that no variables important to their lived experience are missing. We will also discuss with caregivers and youth the acceptability of the CDE set, given that some items in the data element set may be considered sensitive information. We will also ask about the appropriateness of collecting information about patient/family needs without the ability to intervene at all sites. For example, adverse childhood experiences were excluded from the final CDE set due to concerns of collecting the information if some sites could not intervene for positive screens such as through safety planning and provision of accessible mental health resources. We will use the data from qualitative interviews and focus groups to reduce the number of CDE and prioritize CDE as we recognize that the entire preliminary set may lack feasibility for full inclusion in many research studies. Once we have developed a refined list, we plan to pilot test data collection in the context of a research study at a limited number of institutions to identify and correct any pitfalls. Before completing this extensive process, we have not made formal recommendations for incorporation of the CDE in PERC studies but have shared the proposed CDE with PERC for consideration for use in this early stage. We encourage pediatric epilepsy researchers to consider which of these CDE might be important to collect in their own studies and settings moving forward.

Our findings highlight a variety of sociodemographic variables prioritized as important yet excessively difficult to include in the final CDE set. We believe that these elements may be important targets for the development of future validated instruments or measurement techniques. One important example is measurement of English language proficiency. Although validated instruments exist, their length is burdensome to implement in studies not specifically focused on language proficiency.^18^ Novel instruments would likely be relevant not only within pediatric epilepsy research but also to pediatric health equity research more broadly.

Concept Mapping has not been published previously on any topic related to epilepsy and has not previously been used specifically for the development of a CDE set. Therefore, this project also provides information about the feasibility of Concept Mapping to develop CDE, which can be replicated in other pediatric and adult chronic diseases. We also demonstrate the feasibility of using Concept Mapping among epilepsy experts for epilepsy-related topics. Although the methodology is novel in these specific contexts, Concept Mapping has been routinely used in health care, research, and educational initiatives in the past.^19–22^

There are select limitations associated with this study worth noting. Only up to one-third of PERC membership participated in any Concept Mapping activity, although this rate of participation is in line with expectations for research involving groups of health professionals.^10^ The absolute numbers of participants also exceed the minimum numbers of 15 people per activity estimated as necessary for the sufficiency of Concept Mapping data.^10^ There was also limited racial and ethnic diversity of the participants. Of note, PERC does not collect demographic information on its membership roster, so we are unable to compare the diversity of our sample to that of PERC. However, our sample included a greater proportion of individuals with racial identities other than white and a similar proportion of Hispanic/Latino individuals compared with the most recent Child Neurology workforce survey.^23^ In the next phase of the study when we recruit caregivers and youth with epilepsy, we intend to prioritize recruitment of a racially and ethnically diverse sample including some primarily Spanish-speaking participants.

In addition, this CDE set applies only to pediatric epilepsy research studies occurring in the United States, as other countries have different conceptualizations and histories around particular social determinants of health such as race and ethnicity. However, we consider context specificity to be a strength of our CDE. Furthermore, the use of group discussion in the interpretation and finalization phase of Concept Mapping may have introduced groupthink and/or peer pressure, yet also allowed for a greater exchange of ideas, and synthetic and collective decision making, than a purely individualized and anonymized method such as the modified Delphi method.^24^ It is also unknown to what extent this data element set is acceptable to patients and families, although we intend to perform qualitative work to explore patient and family perspectives. Finally, limitations in the implementation of these CDE may exist that cannot be known until they are included in future studies.

## Conclusions

Concept Mapping is a feasible, efficient methodology to foster group consensus among pediatric epilepsy research experts regarding the development of common data elements for health equity in pediatric epilepsy research. This process may serve as a model for development of CDE for health equity in other pediatric and adult chronic medical conditions. Implementation and adoption of CDE will allow more feasible identification and monitoring of health disparities in pediatric epilepsy research studies. Items identified as important yet difficult to measure may require development of novel validated instruments to facilitate evaluation.

## Figures and Tables

**TABLE 1.** Participant Demographics

Demographic Variable	Brainstorming (n = 81)	Sorting (n = 30)	Rating (n = 48)

Profession			
Physician in pediatric neurology or epileptology	54 (67%)	21 (70%)	31 (65%)
Advanced practice provider	0 (0%)	2 (7%)	3 (6%)
Psychologist	11 (14%)	2 (7%)	4 (8%)
Physician in neurosurgery	7 (9%)	2 (7%)	2 (4%)
Psychiatrist	1 (1%)	0 (0%)	0 (0%)
Other*	6 (7%)	3 (10%)	8 (17%)
Did not respond	2 (2%)	0 (0%)	0 (0%)
Years in practice post-training			
0–5 years	22 (27%)	12 (40%)	22 (46%)
6–10 years	19 (23%)	7 (23%)	10 (21%)
11–15 years	15 (19%)	5 (17%)	5 (10%)
16–20 years	13 (16%)	2 (7%)	4 (8%)
Greater than 20 years	11 (14%)	4 (13%)	7 (15%)
Did not respond	1 (1%)	0 (0%)	0 (0%)
Gender identity			
Nonbinary	1 (1%)	0 (0%)	0 (0%)
Woman (transgender)	0 (0%)	1 (3%)	1 (2%)
Woman (cisgender)	49 (60%)	19 (63%)	29 (60%)
Man (transgender)	0 (0%)	0 (0%)	0 (0%)
Man (cisgender)	30 (37%)	9 (30%)	16 (33%)
Other^†^	1 (1%)	0 (0%)	0 (0%)
Did not respond	0 (0%)	1 (3%)	2 (4%)
Race			
American Indian or Alaska Native	0 (0%)	0 (0%)	0 (0%)
Asian	21 (26%)	10 (33%)	12 (25%)
Black or African American	4 (5%)	1 (3%)	1 (2%)
Native Hawaiian or other Pacific Islander	0 (0%)	1 (3%)	3 (6%)
White	45 (56%)	18 (60%)	30 (63%)
Multiracial	7 (9%)	0 (0%)	0 (0%)
Other^‡^	1 (1%)	0 (0%)	1 (2%)
Did not respond	3 (4%)	0 (0%)	1 (4%)
Ethnicity			
Hispanic, Latino, or of Spanish origin	5 (6%)	1 (3%)	5 (10%)
Not Hispanic, Latino, or of Spanish origin	76 (94%)	29 (97%)	43 (90%)
Did not respond	0 (0%)	0 (0%)	0 (0%)

Abbreviation: EEG = Electroencephalography

* Other professions specified: researcher, neuropsychologist, medical assistant, EEG technician.

† Other gender identities specified: one individual wrote "will not answer this question."

‡ Other racial identities specified: Middle Eastern, North African, Jewish, mixed, Hispanic. One individual wrote "I don't know." One individual wrote "will not answer this question."

**TABLE 2.** Proposed Common Data Element Set

Data Elements for Prospective and Retrospective Studies Patient age Race of patient Ethnicity of patient Whether patient is in foster care Whether or not patient has health insurance Health insurance type Whether patient is disabled Whether patient has a co-occurring physical disability in addition to epilepsy Whether patient has a co-occurring neurodevelopmental disability Whether patient is technology dependent (i.e., tracheostomy, gastrostomy tube) Whether family has more than one child with epilepsy or chronic conditions Race of parent(s)/caregiver(s) Ethnicity of parent(s)/caregiver(s) Education level of parent(s)/caregiver(s) Primary language of patient Patient preferred language Primary language of parent(s)/caregiver(s) Preferred language of parent(s)/caregiver(s) Language concordance between parent(s)/caregiver(s) and provider Parent(s)/caregiver(s)' access to the internet Patient access to the internet Ability to participate in telemedicine Zip code Distance from household to epilepsy center (any level) Distance to nearest hospital (pediatric or adult) Measures of annual household income for geographic units larger than neighborhood Whether or not patient has health insurance Neighborhood-level socioeconomic indices Urban versus rural versus suburban area of residence Whether patient is in foster care Whether patient is in school If patient has a 504 plan If patient is involved with Early Intervention services If patient has an Individualized Education Plan Sex of patient
Data Elements for Prospective Studies Only Patient legal status in the USA Parent(s)/caregiver(s) legal status in the USA Family quality of life Access to transportation Household food security Patient/family housing status and security Sexual orientation/sexual identity of patient Gender/gender identity/gender identification of patient
